# Advances in the integration of microalgal communities for biomonitoring of metal pollution in aquatic ecosystems of sub-Saharan Africa

**DOI:** 10.1007/s11356-024-33781-1

**Published:** 2024-06-01

**Authors:** Mary Mulenga, Concillia Monde, Todd Johnson, Kennedy O. Ouma, Stephen Syampungani

**Affiliations:** 1 Department of Biological Sciences, School of Mathematics & Natural Sciences, Copperbelt University, P. O. Box 21692, Kitwe, Zambia; 2 Department of Zoology & Aquatic Sciences, School of Natural Resources, Copperbelt University, P. O. Box 21692, Kitwe, Zambia; 3 Department of Plant & Environmental Sciences, School of Natural Resources, Copperbelt University, P. O. Box 21692, Kitwe, Zambia; 4 Chair-Environment & Development, Oliver R Tambo Africa Research Chair Initiative (ORTARChI), Copperbelt University, P. O. Box 21692, Kitwe, Zambia; 5 Forest Science Postgraduate Program, Department of Plant & Soil Sciences, Plant Sciences Complex, University of Pretoria, Private Bag x20, Hatfield, Pretoria, 0002 South Africa

**Keywords:** Bioindicator, Biosensor, Microalgal e-DNA, Biomonitoring, Artificial intelligence, Citizen science

## Abstract

This review elucidated the recent advances in integrating microalgal communities in monitoring metal pollution in aquatic ecosystems of sub-Saharan Africa (SSA). It also highlighted the potential of incorporating microalgae as bioindicators in emerging technologies, identified research gaps, and suggested directions for further research in biomonitoring of metal pollution. Reputable online scholarly databases were used to identify research articles published between January 2000 and June 2023 for synthesis. Results indicated that microalgae were integrated either individually or combined with other bioindicators, mainly macroinvertebrates, macrophytes, and fish, alongside physicochemical monitoring. There was a significantly low level of integration (< 1%) of microalgae for biomonitoring aquatic metal pollution in SSA compared to other geographical regions. Microalgal communities were employed to assess compliance (76%), in diagnosis (38%), and as early-warning systems (38%) of aquatic ecological health status. About 14% of biomonitoring studies integrated microalgal eDNA, while other technologies, such as remote sensing, artificial intelligence, and biosensors, are yet to be significantly incorporated. Nevertheless, there is potential for the aforementioned emerging technologies for monitoring aquatic metal pollution in SSA. Future monitoring in the region should also consider the standardisation and synchronisation of integrative biomonitoring and embrace the “Citizen Science” concept at national and regional scales.

**Graphical abstract:**

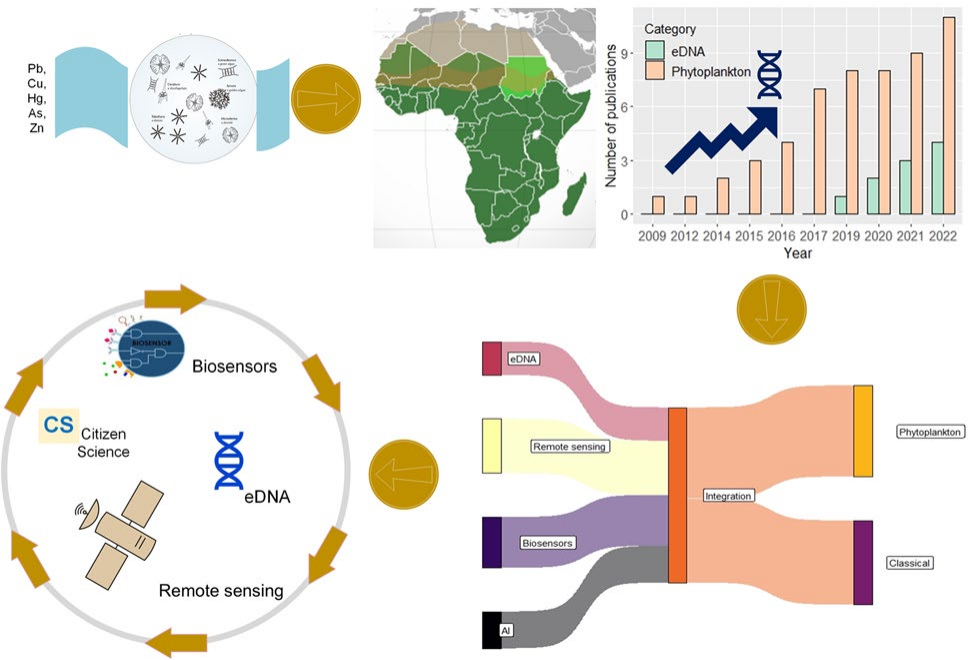

## Introduction

Stream ecosystems play an ecologically significant role in supporting aquatic biodiversity and providing beneficial ecosystem services that sustain the environment and promote human well-being (Limburg 2009; Maes et al. 2020). Stream ecosystem services include fresh water and food provisioning, sediment retention and transport, pollution control, recreation and ecotourism, flood regulation, disease prevention, nutrient cycling, and cultural heritage preservation (MEA 2005). In addition, both stream and riparian systems serve as biodiversity banks for aquatic and semi-aquatic biota as well as developmental stages of several terrestrial fauna such as arthropods, amphibians, and reptiles, among others (Mccabe 2010; Steward et al. 2022). Streams are also critical in transferring nutrients, matter, and energy, thus acting as sources and sinks of pollutants and disease vectors (Limburg et al. 2013; Wohl 2018; Bashir et al. 2020).

However, the negative impact of both natural and anthropogenic pressures has reduced the ability of stream ecosystems to supply aquatic ecosystem services (Khatri and Tyagi 2015). Natural factors such as climate change, droughts, floods, and other natural disasters impact aquatic ecosystems by altering water availability, water quality, and aquatic biodiversity (Costanza et al. 2014; Talbot et al. 2018; Culhane et al. 2019). Anthropogenic factors such as waste disposal, urbanisation, demand for agricultural land and expansion of industrial activities such as metal mining and fossil fuel combustion, and habitat destruction are also significant drivers of deterioration in aquatic ecosystems (Borgwardt et al. 2019; Cormier et al. 2019; Kimirei et al. 2021; Ferreira et al. 2023). Metal pollution of aquatic ecosystems from natural and anthropogenic sources is particularly an environmental and health concern in metal mining regions due to metals being persistent, non-biodegradable, and toxic (Yahya et al. 2018; Ali and Khan 2019; Amoatey and Baawain 2019; Zhou et al. 2020). In the stream ecosystems of Sub-Saharan Africa (SSA), aquatic metal pollution is an ever-growing environmental concern (Biney et al. 1994; Fayiga et al. 2018). There has been a steady accumulation of metals in water, sediment, and aquatic biota in rivers and lakes of SSA, mainly from natural and anthropogenic sources (Zhou et al. 2020; Yabe et al. 2010; Fayiga et al. 2018; Ochieng et al. 2009). In southern Africa, aquatic metal pollution above permissible limits has been reported for stream water and sediments from mining, coal use, and other industrial activities (Ouma et al. 2022; Addo-Bediako et al. 2021). Gerber et al. (2015) and Moyo et al. (2015) noted high Cu, Co, Pb, and Mn in the Olifants River associated with anthropogenic activities and posed a high risk to aquatic biota. Furthermore, Chetty and Pillay (2019) linked the influence of anthropogenic activities to elevated Cr, Cu, Pb, and Zn in Palmiet and Sezela rivers in South Africa’s Kwa-Zulu Natal coupled with high mobility and bioavailability. In the Zambian Copperbelt, Cu, Co, Pb, and Zn mining has impacted the water and sediments of Lake Kariba (Chalumba et al. 2021) and Kafue River with increased ecological risks to aquatic life. In the Katangese Copperbelt of the Democratic Republic of Congo, extreme sediment enrichment with Cu (190.2 mg/kg) and Zn (1117 mg/kg) in the Bumbu River draining Kinsasha has been reported (Kayembe et al. 2018). Banze wa Mutombo (2022) also associated the high pollution of the Mura and Kimpulande tributaries of the Congo River with Cu-Co-As-Cd-loaded mining effluents that increased the vulnerability of aquatic communities.

Metal pollution in West Africa’s aquatic systems has similarly reached alarming levels. Gbogbo and Otoo (2015) reported the detrimental impacts of Cd, As, Hg, and Cu pollution on water, macrophytes, algae, and fish in Ghana’s Sakumo II wetland in the Tema Metropolitan area. According to Ngueyep et al. (2021), Cameroon’s Kadey River tributaries had excess Ni, Fe, Cr, Se, As, and Hg in sediments from artisanal and small-scale gold mining. Tyovenda et al. (2019) reported contamination of water and algae and sediment enrichment with Pb, Hg, Ni, and Fe in River Benue, Nigeria. Despite the relatively low mineral deposit in Eastern Africa, alarming aquatic metal pollution has been reported in Kenya’s gold mining belt (Ngure et al. 2014), Tanzania’s Mara River (Nkinda et al. 2020), Awetu watershed in Ethiopia (Astatkie et al. 2021), and Namukombe stream in Uganda (Omara et al. 2019). For instance, Ngure et al. (2014) noted high Hg (355 mg/kg) in fish, while Astatkie et al. (2021) recorded stream sediment contamination with Pb (2,000 mg/kg), As (623 mg/kg), and Cr (375 mg/kg). Based on the representative studies above, there is sufficient evidence of aquatic metal pollution thus, raising the need for monitoring of aquatic ecosystems across SSA.

**Fig. 1:**
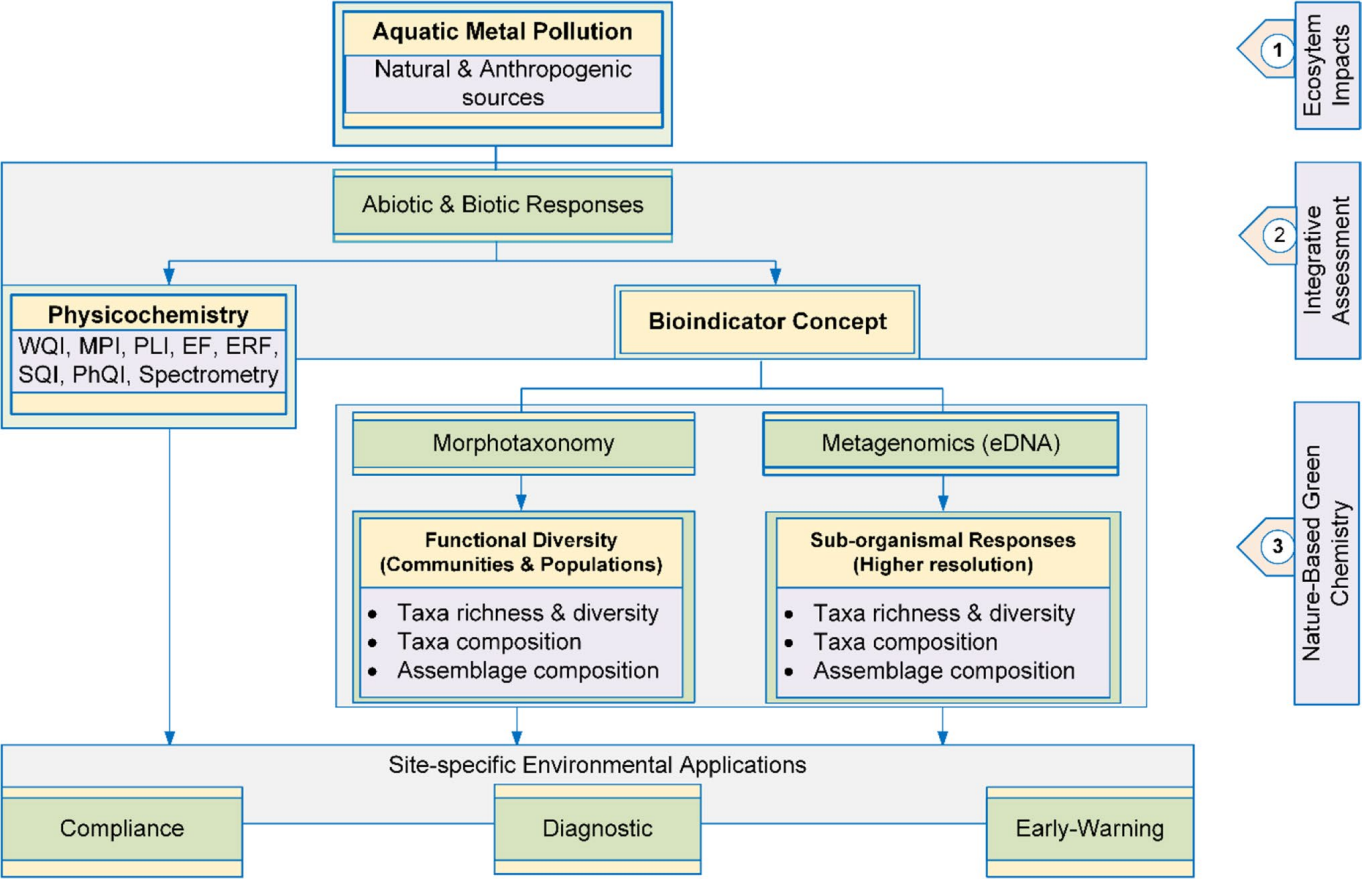
Conceptualising integrative monitoring of aquatic metal pollution in stream ecosystems

One of the approaches that can be employed to effectively monitor and assess the magnitude of anthropogenic and natural impacts on stream ecosystems is the bioindicator concept, which utilises sentinel aquatic biota (Lazorchak et al. 2003; Schwacke et al. 2013; Parmar et al. 2016). Bioindicators reflect the bioavailable fractions of pollutants and hence are of potential ecotoxicological significance (Hamza-Chaffai 2014; Lamare 2019; Kumari and Paul 2020) Based on the targeted outcome, three categories of bioindicators commonly used for monitoring environmental health include early warning, compliance, and diagnostic indicators (Hamza-Chaffai 2014). “Early-warning bioindicators” signify the impending deterioration of ecosystem health. Deviations from the acceptable aquatic environmental conditions are detected by “compliance indicators” while the “diagnostic bioindicators” reflect the causes for the deviations from the expected ecological conditions of the aquatic ecosystem (Sumudumali and Jayawardana 2021). Figure 1 illustrates the bioindicator concept of aquatic metal pollution biomonitoring that utilises the compliance, diagnostic, or early-warning aspects of indicator species or communities.

Aquatic microalgal communities have been utilised to monitor the ecosystem health in lotic environments (Yilmaz et al. 2021; Feisal et al. 2023). The vital ecological role of microalgal communities has been recognised through continuous surveillance to establish their status in the aquatic environment (Li et al. 2021; Thompson and Carstensen 2023). The ability of microalgae to accumulate high levels of pollutants, their relative sessile nature, ease of sampling, ease of culture in the lab, and their trophic importance as primary producers in the aquatic food web have positioned them as suitable bioindicators of metal pollution (Zhou et al. 2008; Parmar et al. 2016; Kumari and Paul 2020). Freshwater microalgae occur either as planktonic, which dominate the pelagic zone, or the benthic forms associated with substrates such as sediments, rocks, macrophytes, mud, and organic debris (Bellinger and Sigee 2015).

Globally, the use of microalgae for biomonitoring as early-warning signals has been widely documented. For instance, in Europe, Dokulil et al. (2016) documented long-term historical biomonitoring using microalgae responses and community composition in the extensive Danube River hydro system. Furthermore, biomonitoring of metal pollution in the transboundary Danube River delta aquatic complex reported high concentrations of bioavailable Ni, Cd, and other potentially toxic elements (Burada et al. 2015; Simionov et al. 2021; Calmuc et al. 2021). Metal pollution trends were also observed in the southeastern Brazil river basin impacted by metal contamination from the Mariana dam failure, with increased Hg bio uptake by microalgae (Marques et al. 2022). Silva et al. (2022) further reiterated the significance of using morphological and taxonomic responses of microalgae as bioindicators to environmental changes in river basins of southern Brazil. In India, microalgal communities in the tropical freshwater Godavari River, Cu, and Zn exhibited lethal effects at high concentrations for the dominant cyanobacteria and chlorophytes (Chakraborty et al. 2010). Feng et al. (2021) also noted the detrimental impacts of metal pollution on the microalgal community structure, with certain microalgal species being more sensitive to the bioavailable metals in the Yangtze River in China. In the Sefid Rud River, Iran, changes in microalgal assemblages were suitable bioindicators of environmental variability and corresponded to physical and chemical changes in the south Caspian Sea catchment (Ramezanpour et al. 2014).

Microalgal communities have also been used to monitor metal pollution in Africa’s stream ecosystems. In West Africa’s Niger River system, Ezewudo et al. (2021) noted weak to high potential ecological risks to aquatic communities, including microalgae, from As, Cd and Hg contamination. In the Cameroon Centre Region, the spatial-seasonal changes in algal densities in the streams of the Sanaga lotic system network draining urban and industrial settlements corresponded to changes in the aquatic physicochemical environment (Pascale 2023). According to Mangadze et al. (2019a), several ecological health studies on southern Africa’s rivers have applied benthic diatoms for biomonitoring. Dalu et al. (2014) noted a direct response of microalgal communities to changes in the physicochemical environment of the Kowie system riverine-estuarine continuum in South Africa’s Eastern Cape. Recent studies on South Africa’s urban Molopo River depicted anthropogenic Cu, Cr, Zn, and Pb sediment contamination with potentially deleterious ecological impacts on the benthic algal and macrofauna communities (Mohajane and Manjoro 2022). Additionally, the diatom-based biomonitoring tools (e.g., the “South African Diatom Index (SADI)” and the “Benthic Diatom Index (BDI)”) have been used to detect and quantify the magnitude of natural and anthropogenic impacts on stream ecosystems (Lang et al. 2013; Harding and Taylor 2014; Sirunda et al. 2021).

Microalgae have the potential to be integrated into conventional monitoring programs as complementary tools to increase the resolution in detecting sub-lethal contamination and thus serve as early-warning bio-systems (Cid et al. 2012; Bae and Park 2014). Despite the potential of integrating microalgal communities in the biomonitoring of aquatic ecosystems, this approach remains one of the least explored alternatives to sustainable management of freshwater ecosystems in SSA (Lemley et al. 2016). Therefore, this review seeks to (1) provide insights into recent advances in the integration of microalgae in biomonitoring metal pollution in the SSA lotic systems, (2) highlight the potential of integrating microalgal as bioindicators in the emerging technologies for monitoring aquatic metal pollution of lotic systems, and (3) identify research gaps and suggest directions for further research in the integrating microalgae as bioindicators of metal pollution in lotic systems of SSA.

## Methodological approach

### Scope of literature search

To ensure that high-quality and relevant articles were selected, our review defined explicit inclusion criteria outlined in Cornelissen et al. (2009). The literature search included articles addressing advances in integrating microalgae for biomonitoring metal pollution in stream ecosystems draining metal-mining landscapes of SSA. The search was restricted to original research, written in English, from articles published between January 2000 and June 2023 to identify ‘gold-standard’ literature on stream biomonitoring of metal pollution with a focus on microalgae as bioindicators.

**Fig. 2:**
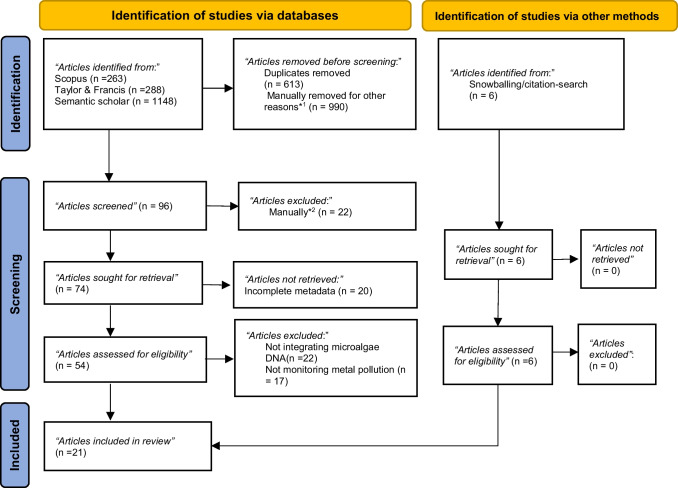
The PRISMA protocol for identifying, screening, and including literature for the review

The article selection process aims to identify the original research papers that present clear evidence of the study objectives (Syeed et al. 2023). Page et al. (2021) preferred reporting items for systematic review and meta-analysis (PRISMA) protocol was followed to ensure a comprehensive and well-defined strategy for the identification, screening, and inclusion of articles for review (Fig. 2). Reputable academic databases, SCOPUS, Taylor and Francis, and Semantic Scholar were searched for authentic articles (Kitchenham and Charters 2007). Furthermore, snowballing or citation-searching from “gold-standard” literature was used to identify more articles for preliminary screening (Wright et al. 2014).

The literature search was conducted based on the article title, abstract, and keywords using key terms such as “bioindicator”, “aquatic biomonitoring”, “metal mining”, “aquatic pollution”, “algae”, “aquatic ecosystems”, “sub-Saharan Africa”, “Africa”, “e-DNA”, “environmental DNA”, “metagenomics”, “microalgae”, “Biosensors”, “Remote sensing”, and “Citizen science”. From each article included, the following information was extracted: (i) title, (ii) authors, (iii) publication year, (iv) regional distribution (v) main objective, (vi) methods (physical, chemical, biological) (vii) indicator organism(s), (viii) environment (sediment, water, biota), (ix) pollutant(s) (x) microalgal metrics used, and (xi) key findings, gaps, or recommendations.

### Streamlining article evaluation and selection

#### Quality assurance and bias reduction

To remove bias in the first stage of the search, the authors searched independently in the digital databases using search terms with slightly varying synonyms to maximise the extraction of articles from the global search. This initial search was followed by the within-results search, where the authors used the same filter criteria specifying the period, the document type, the region of study, and the field of study. In the second stage, the authors verified the extracted articles’ metadata for completeness and originality. Articles that fulfilled the quality assurance process were included for further synthesis.

#### Article processing

The results from the databases were downloaded and imported into the Mendeley reference software (Mendeley Ltd). The following metadata was checked and updated where necessary for each article: author(s), title, year of publication (and month), volume, page numbers or article number, abstract, keywords, and DOI, if available. However, articles for which pertinent metadata items such as author, title, or publication year that were missing were further excluded from the list.

#### Exclusion process

An automated keyword-based search was used to explore the database and extract relevant research articles (Beecham et al. 2008). The terms were searched in the article titles, abstracts, and keywords. The exclusion criteria for out-of-scope articles were principally based on the following aspects: (1) studies outside freshwater systems, e.g., oceans and seas; (2) other bioindicator categories used, e.g., non-photosynthetic bacteria, marine plankton, freshwater zooplankton, macrophytes, macroalgae, macroinvertebrates, and vertebrates; (3) clinical and laboratory biomonitoring studies, e.g. humans and wild and domestic animals using water resources; and (4) studies involving aquatic ecosystem pollutants other than metals. Furthermore, manual removal was conducted to ensure that only relevant and complete articles were included in the final review process (Petticrew and Roberts 2008).

### Bibliometric analysis

**Fig. 3:**
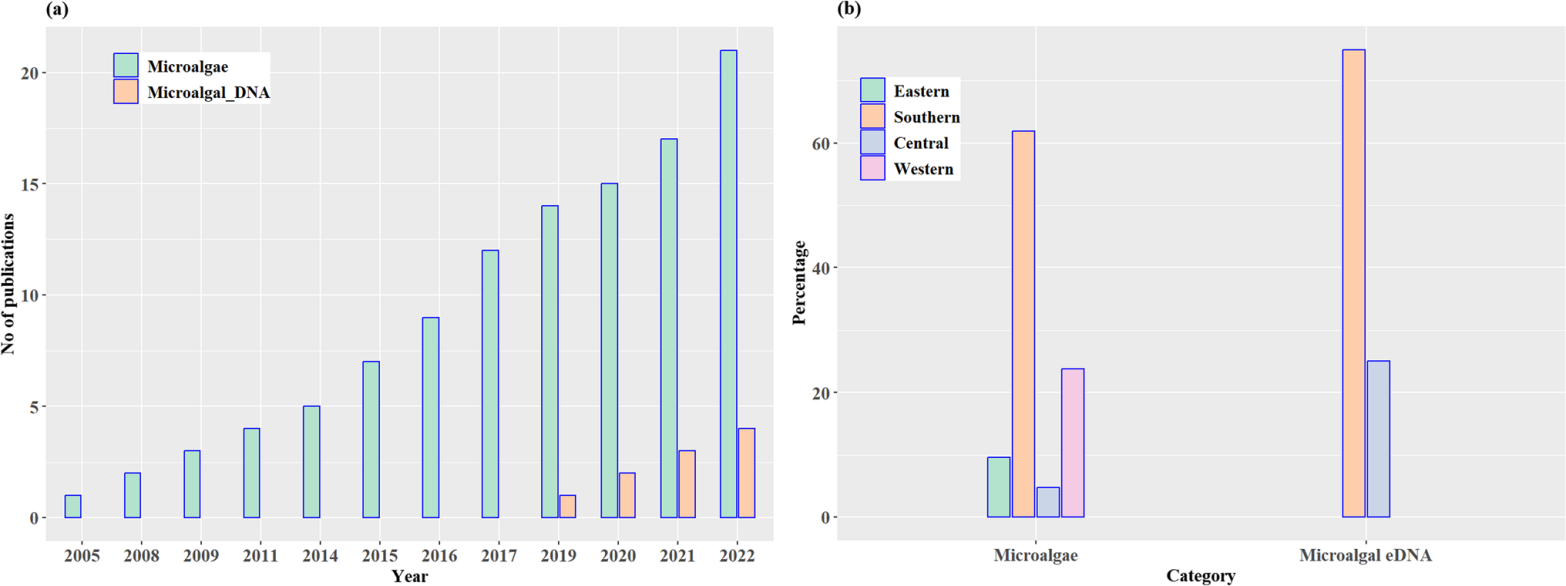
**a** Distribution of publications by year and research focus and **b** regional proportions of microalgae and environmental DNA (eDNA) integration in the monitoring of metal pollution in aquatic ecosystems of sub-Saharan Africa

A bibliometric analysis of the extracted information was conducted to classify articles based on the year of publication, authors, region, main objective(s), bioindicator type, environmental matrix, pollutant(s), methods, and the microalgal metric(s). Following the PRISMA filtering protocol, the review included 21 articles (15 from the digital scholarly databases and six from snowballing/citation search) relevant to the research area, geographical location, and study period. From Fig. 3a, between January 2000 and June 2023, there was a notable general cumulative 95% increase in the studies incorporating different microalgal taxa in monitoring metal pollution in aquatic ecosystems in the SSA. This indicates a growing interest in incorporating microalgal taxa in aquatic biomonitoring.

Generally, between January 2000 and June 2023, studies indicate that only South Africa, Namibia, and the Democratic Republic of Congo (DRC) integrated microalgae and microalgal-based eDNA, respectively, in aquatic biomonitoring for metal pollution. Sub-regionally, only 24% of the countries in West Africa, 10% in Eastern Africa, 25% in Central Africa, and 75% in Southern Africa conducted microalgal-based biomonitoring of metal pollution in streams. However, microalgal-eDNA integration in aquatic metal pollution biomonitoring is still in its infancy in SSA, with only Central and Southern Africa accounting for 25% and 75% of aquatic-based research to monitor metal pollution (Fig. 3b). The integration of microalgal-eDNA method in aquatic metal pollution biomonitoring in SSA was first documented by Jordaan et al. (2019), who noted a 6% variability in bacterial community composition and diversity from the anthropogenic Co, As, Cr, Ni, and U pollution in the rivers within the lower Wonderfonteinspruit catchment of South Africa. Since then, the eDNA approach has been seen as a potential approach to accelerate aquatic biomonitoring by supplementing traditional taxomorphological monitoring in the SSA landscape (Perry et al. 2022).

### Bioindicator taxa and environmental assessment

**Fig. 4:**
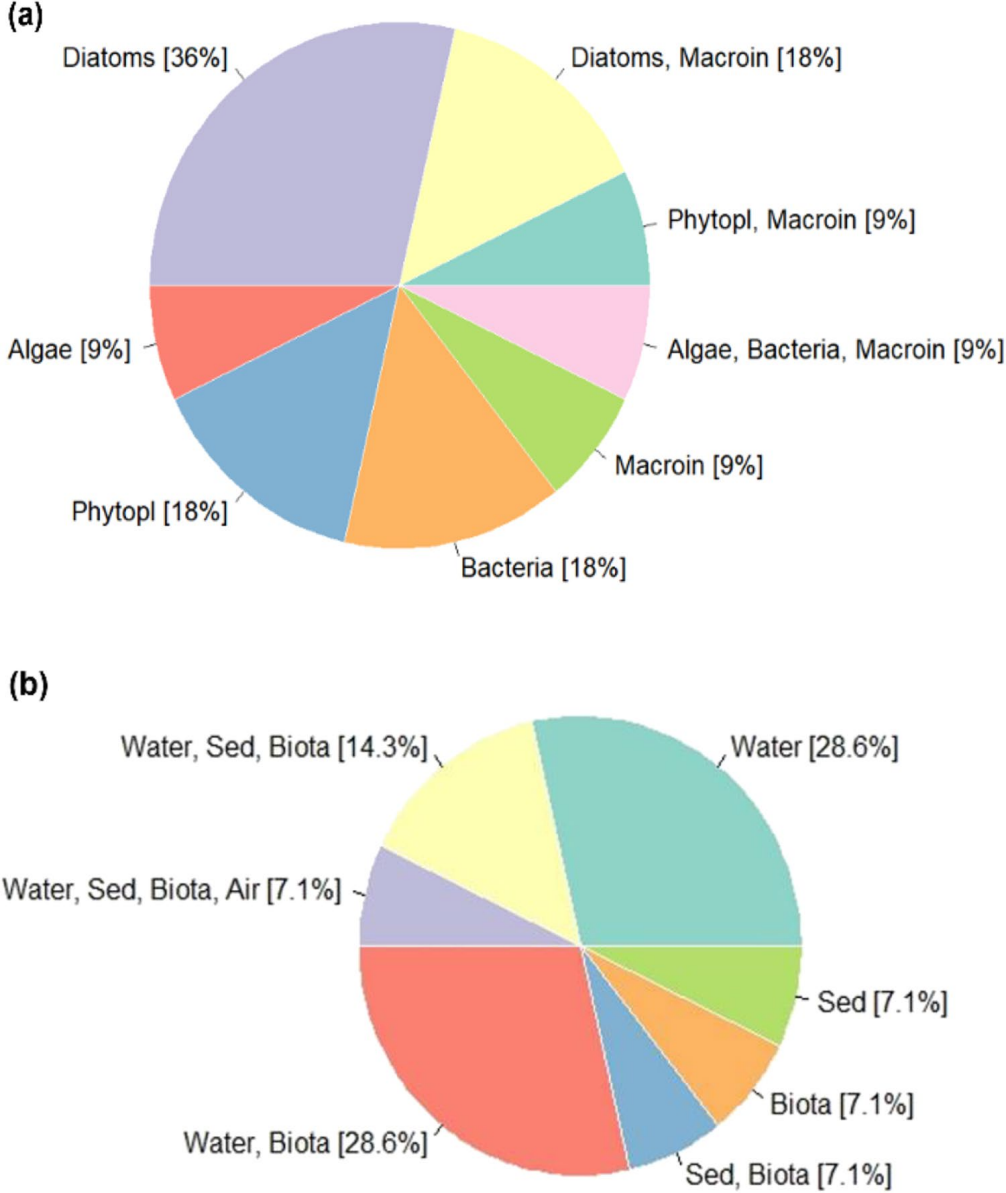
**a** Bioindicator integration in aquatic metal pollution biomonitoring in SSA; **b** environmental matrices investigated for metal pollution. Macroin macroinvertebrates, Phytopl phytoplankton, Sed sediment

The diatoms are the single most preferred microalgal bioindicator taxon (36%) and are also used with benthic macroinvertebrates (18%) to monitor aquatic metal pollution. Considering their specificity and sensitivity to ecological changes in aquatic ecosystems, diatoms have been widely employed to detect perturbations in stream water quality (Lobo et al. 2016; Mangadze et al. 2017). The preference for both taxa could be attributed to their stationary and benthic nature, which makes them suitable for recording long-term pollutant trends compared to the instantaneous physicochemical methods that only consider a “snap-shot” of the environmental water quality (Beyene et al. 2009; Hattikudur et al. 2014). Other single-use taxa of microalgae, including cyanobacteria, had equal preferences (18%), while “algae” and macroinvertebrates comprised 9% each as the bioindicators used to assess metal pollution (Fig. 4a).

Regional studies elsewhere, for example, in North America (e.g., Smucker et al. 2018) and Asia (e.g., Chon et al. 2013), have similarly integrated algae and macroinvertebrates as well as microbial communities to monitor metal pollution of freshwaters while leveraging on the producer–consumer trophic changes as indicators of disturbances at catchment scale. Furthermore, Respondek et al. (2022) integrated mosses and microalgae in monitoring metal pollution in surface water in the smelter area of Ozimek, Poland. They observed diatom taxa as the dominant algal group that indicated responses to metal stress, e.g., the metal-tolerant *Achnanthidium* sp and *Mayamaea* sp dominated up to 99% of the algal communities, and served as excellent bioindicators of metal contamination. In addition, Pandey (2020) compared green algae*,* cyanobacteria, and diatom species and noted an increased relative taxa abundance, indicating increased tolerance to metal pollution. Moreover, increased lipid production and cell-wall teratologies in diatoms, also indicated by Lavoie et al. (2012), were observed under Cu, Cd, Zn, and Pb stress. Pandey and Bergey (2018) also found that diatoms-dominated periphyton biofilms were excellent indicators of metal pollution, thus showing the utility of periphytic diatom communities as an effective tool for biomonitoring of aquatic metal pollution. Gbogbo and Otoo (2015) used the biomonitoring potential of algae, among other bioindicator biota of an urban wetland system in Ghana, to determine the magnitude of metal pollution algae accumulated up to 12 mg/g Cd. Similarly, Leguay et al. (2016) and Solak et al. (2020) reiterated the importance of complementing physicochemical assessment techniques with diatom-dominated biofilm-based proxies, diatom indices (e.g., the Pampean Diatom Index and Specific Pollution Index) to monitor metal contamination in aquatic systems.

In Fig. 4b, water is the most frequently assessed abiotic matrix (28.6%) and in combination with different bioindicators in the same proportion for assessing metal contamination. Studies by Dalu et al. (2017, 2022a) and Tyovenda et al. (2019) included stream sediment plus water, diatoms, algae, and benthic macroinvertebrates in the evaluation of metal pollution to obtain a three-way health status of the aquatic ecosystem. In addition, Mangadze et al. (2017) incorporated a fourth dimension of atmospheric contribution to stream ecosystem metal pollution to assess the potential of using diatoms as suitable bioindicators of ionic metal pollution along a South African temperate river system.

### Application of microalgae for biomonitoring tropical stream ecosystems of SSA

**Fig. 5:**
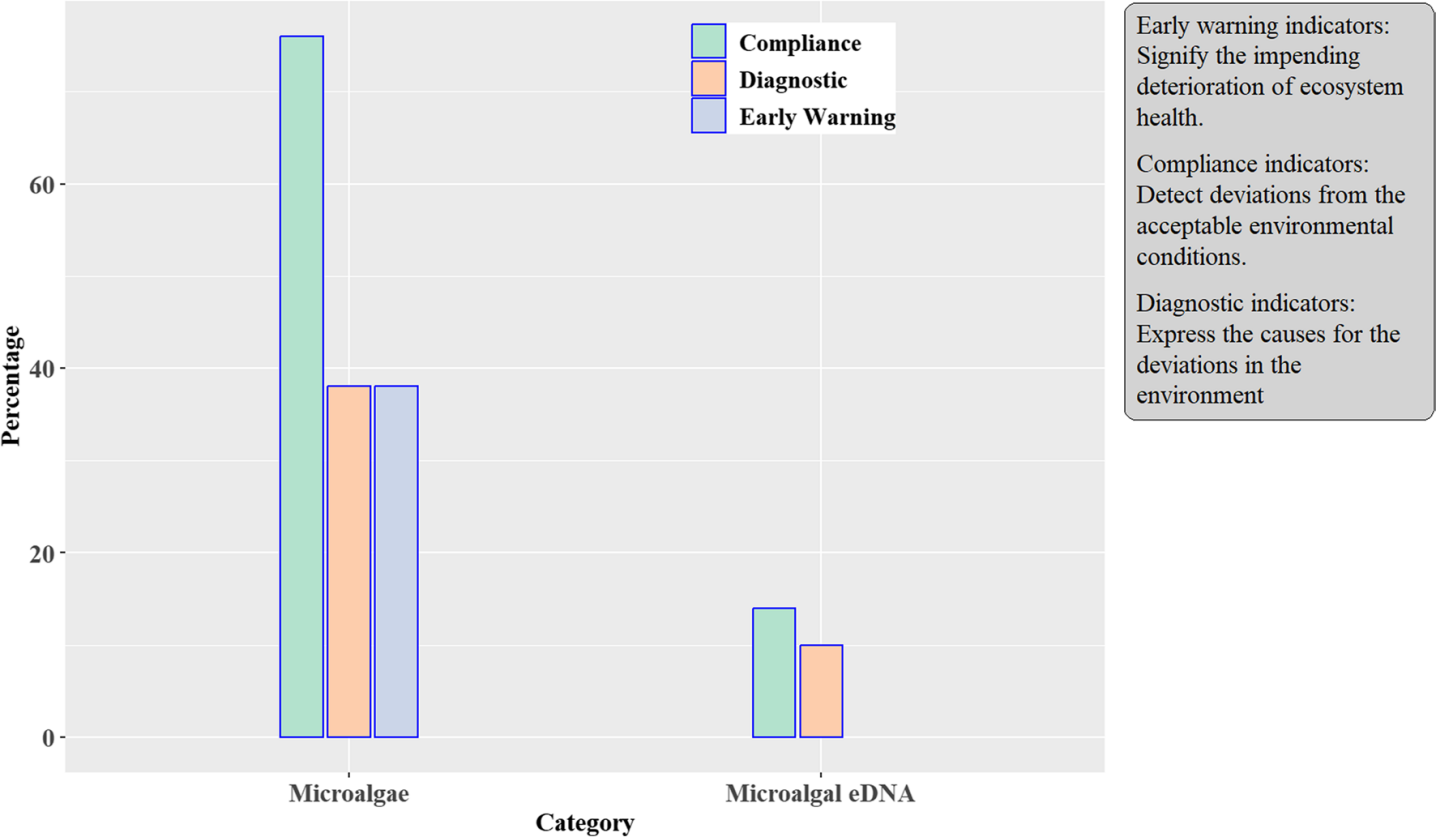
Application of the microalgal taxa in the integrated monitoring of the environmental conditions of aquatic ecosystems exposed to metal pollution in SSA

In Fig. 5 and Table 1, three studies (by Jordaan et al. 2019 Laffite et al. 2020 Perry et al. 2022) incorporated the microalgal-eDNA to check for environmental compliance with the established national or international guidelines for metal contaminant levels in freshwater aquatic environments. Jordaan et al. (2019) and Pereira‐da‐Conceicoa et al. (2021) used eDNA as a diagnostic tool to determine the causes of deteriorating water quality and changes in microbial communities in South African river catchments. However, no study used microalgal-eDNA for early warning of aquatic ecosystem change, making this a potential area for future research. Most studies (76%) employed various microalgal taxa responses for compliance monitoring, followed by diagnostic and early-warning functions, each at 38% (Fig. 5).

**Table 1:** Environmental applications of microalgal taxa as bioindicators for monitoring of aquatic metal pollution in stream ecosystems of SSA

No	Author(s), date, title	Main objective(s)	Application (C, compliance;D, diagnostic;E, early warning)			Related studies
1	Oberholster et al. (2017): “River catchment responses to anthropogenic acidification in relationship with sewage effluent: An ecotoxicology screening application.”	(a) “Establishing the current state of the aquatic ecosystems affected by acidification by using different aquatic indicator organisms as screening tools.” (b) “Determining the response of the system to a mixture of AMD and acid precipitation in tandem with domestic sewage effluent and agriculture activities at sub-catchment scale.”	C	D		Bermanec et al. (2018)
2	Dalu et al. (2022a): “Land use effects on water quality, habitat, and macroinvertebrate and diatom communities in African highland streams.”	“Evaluating the impacts of land use on biotic components. This study analysed the diatom and macroinvertebrate community composition of the Eastern Highlands (Zimbabwe) streams to assess the main spatial diatom and macroinvertebrate community variances and how environmental variables and spatial factors influence community composition”	C	D		Mwedzi et al. (2020)
3	Mangadze et al. (2017): “Use of Diatom Communities as Indicators of Conductivity and Ionic Composition in a Small Austral Temperate River System.”	“Determining the application of benthic diatoms as effective and reliable indicators of ionic composition and conductivity in different stream order categories.”	C		E	Deng et al. (2012)
4	Tyovenda et al. (2019): “Assessment of Heavy Metal Pollution of Water, Sediments and Algae in River Benue at Jimeta-Yola, Adamawa State, Nigeria”	“Assessing heavy metal pollution in water, sediments and Algae in the upper region of River Benue at Jimeta-Yola, Adamawa state, Nigeria.”	C			Dora et al. (2021)
5	Dalu et al. (2017): “Variation partitioning of benthic diatom community matrices: Effects of multiple variables on benthic diatom communities in an Austral temperate river system.”	(1) “Exploring and describing the diatom species assemblages of the Bloukrans River system covering the primary environmental gradients in an urbanised and agricultural intensive Bloukrans River system, Eastern Cape, South Africa, with the view of improving and understanding diatom-based water quality assessment systems.” (2) “Assessing the importance of analysed variables of the water column, sediment, and physical properties of the sites and their contributions in explaining diatom community structure and species richness variation in the system.”	C	D		Ferreira da Silva et al. (2009)
6	Oberholster et al. (2016): “Algal assemblage responses to acid mine drainage and steel plant wastewater effluent up and downstream of pre and post-wetland rehabilitation”	(1) “Employing freshwater algae to differentiate between pre and post-wetland rehabilitation conditions” (2) “Determined whether algae can be used as reliable bioindicators for wetland enlargement rehabilitation measures”	C	D	E	Ali and Abd el-Salam (1999)
7	Beyene (2009): “Comparative study of diatoms and macroinvertebrates as indicators of severe water pollution: Case study of the Kebena and Akaki rivers in Addis Ababa, Ethiopia”	“Investigating the responses of diatom and macroinvertebrate community structures to major environmental gradients in three disturbed and stressed rivers in Addis Ababa, Ethiopia”	C	D	E	Blanco and Bécares (2010)
8	Hena et al. (2022): “Distribution of heavy metal and phytoplankton in Calabar river port terminals, Calabar, cross river state, Nigeria”	“Assessing the seasonal variation of heavy metals and the distribution of phytoplankton in the Calabar River Port terminals”	C		E	Pandey and Bergey (2018)
9	Ugbeyide and Ugwumba (2021): “Water quality and phytoplankton as indicators of pollution in Ibuya river”	“Determining the pollution index of Ibuya River by assessing the water quality and phytoplankton structure of the ecosystem”	C	D		Bermanec et al. (2018)
10	Mangadze et al. (2015): “Epileptic diatom flora in contrasting land-use settings in tropical streams, Manyame Catchment, Zimbabwe”	“Evaluating the response of stream diatom assemblages to changes in water quality in different land-use settings”	C	D		Tapia (2008)
11	Bere et al. (2014): “The application and testing of diatom-based indices of stream water quality in Chinhoyi Town, Zimbabwe”	“Testing the applicability of foreign diatom-based water quality assessment indices to urban streams in Zimbabwe, with the view of stimulating research to develop improved diatom-based approaches for assessing the ecological integrity of lotic systems in Zimbabwe”	C		E	Leguay et al. (2016)
12	Jordaan et al. (2019): “An Integrated Insight into the Response of Bacterial Communities to Anthropogenic Contaminants in a River: A Case Study of the Wonderfonteinspruit Catchment Area, South Africa”	“Characterizing bacterial communities in the lower Wonderfonteinspruit and their response to various contaminant sources using eDNA”	C	D		Ancion et al. (2010)
13	Laffite et al. (2020): “Impact of anthropogenic activities on the occurrence and distribution of toxic metals, extending-spectra β-lactamases and carbapenem resistance in sub-Saharan African urban rivers”	(i) “Quantification of β-lactam resistance genes (blaSHV, blaCTX-M, blaNDM, blaKPC, blaOXA-48, blaVIM, blaIMP) by quantitative PCR;” (ii) “Determination of the impact of Cr, Co, Ni, Cu, Zn, Cd, Pb, Hg on bacterial communities”	C			Ancion et al. (2010); Li et al. (2018)
14	Pereira‐da‐Conceicoa et al. (2021): “Metabarcoding unsorted kick-samples facilitates macroinvertebrate-based biomonitoring with increased taxonomic resolution while outperforming environmental DNA”	“Use of several DNA-based survey methods for water quality and biodiversity assessment in South Africa”		D		Ancion et al. (2010); Li et al. (2018)
15	Perry et al. (2022): “Challenges to Implementing Environmental-DNA Monitoring in Namibia”	“Testing field and laboratory eDNA protocols (aqueous and sediment samples) in a range of semi-arid freshwater ecosystems in central and northern Namibia (using gathered eDNA data from a broad-suite of organisms at multiple trophic levels (including algae, invertebrates and bacteria)”	C			Harrison et al. (2019); Bunholi et al. (2023)
16	Bere et al. (2016): “Variation partitioning of diatom species data matrices: Understanding the influence of multiple factors on benthic diatom communities in tropical streams”	“To explore the relative impact of metal pollution and hydromorphological alterations, in addition to nutrient enrichment and organic pollution, on diatom taxonomic composition with the view to improve stream diatom-based water quality inference models”	C		E	Kock et al. (2019); Lee et al. (2021)
17	Kaonga et al. (2008): “Levels of cadmium, manganese and lead in water and algae; *Spirogyra aequinoctialis*”	“To assess the ability of filamentous green algae; *Spirogyra aequinoctialis,* to accumulate manganese, cadmium and lead from water”	C			Rajfur et al. (2010); Vetrivel et al. (2017)
18	Dalu et al. (2022b): “Community structure and environmental factors affecting diatom abundance and diversity in a Mediterranean climate river system”	“The study sought to detect diatom community patterns and to understand the processes that cause these structures in an Austral Mediterranean river system among different months and river sections”		D	E	Tolotti et al. (2019); Rivera et al. (2020); Masouras et al. (2021)
19	Gbogbo and Otoo (2015): “The concentrations of five heavy metals in components of an economically important urban coastal wetland in Ghana: public health and phytoremediation implications.”	“The study sought to present the levels of five heavy metals, namely Cd, As, Hg, Pb and Cu, in some biotic and abiotic components of an economically important urban coastal Ramsar site in Ghana”	C			Marcel et al. (2017); Solak et al. (2020)
20	Awofolu (2005): “Determination and seasonal variation of heavy metals in algae and sediments in sewers from industrial areas in Lagos State, Nigeria”	“To determine the level of heavy metals (Cd, Pb, Cu and Zn) in algae and sediments in sewers from industrial areas of Lagos state”	C			Tyovenda et al. (2019)
21	Ogoyi et al. (2011): “Determination of Heavy Metal Content in Water, Sediment and Microalgae from Lake Victoria, East Africa”	“To assess the levels of heavy metals in water and sediment from Lake Victoria and how this relates to bioaccumulation of the pollutants in microalgae. The study also determined how the level of heavy metal pollution varies with dry, short rain and long rain seasons”	C		E	Chmielewská and Medved’ (2001); Ismail and El Zokm (2023)

Several studies combined more than one environmental application of algal communities to test for compliance, diagnosis, or early warning to evaluate the overall integrity and potential ecological risks for the respective aquatic ecosystems investigated. Despite its environmental importance, only 30% of the studies used biomonitoring as an early-warning tool, while 40% employed biomonitoring for diagnostic purposes. Nevertheless, 91% of the studies were targeted to determine environmental compliance of anthropogenic activities that introduce metal contaminants to the aquatic environment against the set effluent discharge standards in the pro-active management of aquatic ecosystems in SSA. Table 1 highlights the environmental applications (compliance, diagnostic, and early warning) of microalgal communities based on the main objectives of the reviewed studies. Algal communities are helpful for compliance, diagnostic, or early-warning biomonitoring since they reflect long-term changes in stream water quality (Mangadze et al. 2016) (Table 1). Ugbeyide and Ugwumba (2021) assessed the physicochemical and biological status of the Ibuya River in Nigeria, which was impacted by anthropogenic pollution. Cd (0.003 mg/L) and Pb (3.5 mg/L) levels exceeded permissible limits for surface water quality, while the lower species richness and composition, dominated by Bacillariophyceae, reflected a lotic system impacted by allochthonous pollution. Oberholster et al. (2016) observed increased algal species diversity caused by improved downstream water quality during the rehabilitation of the Grootspruit wetland, South Africa, impacted by acid-mine drainage. The trends concur with a previous study by Ali and Abd el-Salam (1999) that noted changes in the dominance of microalgal species *Cyclotella* and *Nitzschia* (Bacillariophyta), *Actinastrum* and *Scenedesmus* (Chlorophyta), and *Oscillatoria* sp (Cyanophyta).

Furthermore, in the Macedonian Maidanska River, the bioconcentration and biomineralisation of Cu, As, Cr, Se, and Cs were observed in *Audouinella* sp, while the high bioaccumulation of Ba (3 mg/g) and intracellular biomineralisation were evidenced in Spirogyra sp. thereby positioning these algal species as a biological pathfinder for acid-mine drainage deposits (Bermanec et al. 2018). Water and sediment chemistry, including nutrient and metal pollutants, largely influence the stream algal community composition. Dalu et al. (2017) explored the influence of anthropogenic impacts on diatom communities and noted the dominance of pollution-tolerant taxa in an austral temperate stream in South Africa. The tolerance and morphological changes (teratologies) on epilithic diatom communities have also been employed to monitor and quantify the biological effects of metal stress from an abandoned Coval da Mo mine drainage (Ferreira da Silva et al. 2009). The findings agree with Pandey and Bergey (2018), who correlated non-taxonomical parameters, including teratologies and lipid bodies, to indicate metal toxicity and recovery in fluvial systems. Diatom indices, including the GDI (Generic Diatom Index), BDI (Biological Diatom Index), and TDI (Trophic Diatom Index), were successfully employed to monitor the Dongjiang River in China with BDI and GDI showing an apparent response to water quality changes (Deng et al. 2012). Diatoms have also been incorporated in multispecies biomonitoring of the temporal variability of metal pollution in Nigeria’s Calabar River (Hena et al. 2022) and Kebena-Akaki Rivers, Ethiopia (Beyene et al. 2009). In both studies, a significant response was observed between the algal community structure and metal concentrations. Mangadze et al. (2017) similarly reflected the role of diatom assemblages as bioindicators of metal pollutants (e.g., As, Zn, Cu, and Cr), particularly on low pollution tolerant species such as *Fragilaria*, *Cyclostephanos*, and *Gyrosigma* transition to high pollution tolerant forms (e.g., *Nitzschia* and *Gomphonema*). This observation is also supported by findings in Dalu et al. (2022b), where changes strongly influenced the structure of diatom communities in water and sediment quality due to the presence of metal contaminants such as B, Cu, and Fe in the Krom River system of the western cape, South Africa. Microalgal communities have also been used to indicate metal pollution in lacustrine systems. For instance, Ogoyi et al (2011) determined metal concentrations (Zn, Pb, Cd, Cr, and Hg) in algal communities alongside water and sediment as an integrative aquatic ecosystem assessment approach.

Integrating microalgal communities into molecular tools for compliance, diagnostic, or early-warning monitoring of streams in mining regions of SSA is also ongoing. Jordaan et al. (2019) studied the influence of anthropogenic pollution on the structure and function of aquatic bacterial communities, using 16S rRNA as a proxy indicator, in South Africa’s Wonderfonteinspruit river catchment. Pereira-da-Conceicoa et al. (2021) demonstrated the merits of incorporating eDNA into existing aquatic biomonitoring metrics with the potential of recovering more diversity and a higher resolution. The ecological advantages of integrating eDNA studies in aquatic biomonitoring above are also evident in other global investigations. Li et al. (2018) noted that the operational taxonomic units of molecular e-DNA data can predict up to 79% of aquatic pollution. Ancion et al. (2010) used 16S rRNA gene libraries to examine the impact of Cu, Zn, and Pb on bacterial communities embedded in freshwater biofilms and recorded higher sensitivities, thereby confirming their potential role as compliance indicators of stream health.

### Method integration and environmental and biological metrics used for assessment of metal pollution in aquatic ecosystems of SSA

All the reviewed microalgal-based works combined physicochemical and biological techniques to investigate metal pollution, possibly to enhance the detection of contaminants and their impact on biota (Torrisi et al. 2010). Several environmental and biological indices were used to quantify the magnitude of the impact of metal pollution, including enrichment and contamination factors, pollution indices, species richness, and diversity indices (Bere et al. 2016; Dalu et al. 2022a; Mangadze et al. 2019b; Ugbeyide and Ugwumba 2021) (Table 2). According to Lobo et al. (2016), biotic indices such as Beck’s index and Renberg’s “Index B” developed from the relative abundances of bioindicator species have been employed for biomonitoring of streams and other aquatic ecosystems. The determination of the physicochemical water quality coupled with the estimation of aquatic biodiversity based on biotic indices has been used to infer the ecological health status and as “early-warning” indicators of aquatic ecosystem health changes (Bellinger and Sigee 2015; Forio and Goethals 2020). Geochemical indices such as contamination factor (CF), enrichment factor (EF), geo-accumulation index (I_geo_), and pollution load index (PLI) were used to evaluate the occurrence and magnitude of pollution in SSA streams receiving metal(loid) contaminants (Tyovenda et al. 2019; Hena et al. 2022). Changes in algal community composition, abundance, PLI, and metal pollution index (MPI) have also been used to assess aquatic metal pollution stress in aquatic communities of Egypt’s Alexandria coast (Ismail and El Zokm 2023).

**Table 2:** Integrated bio-physico-chemical monitoring of stream ecosystems environment using various metrics for quantification of impacts of metal pollution. The reference numbers refer to studies summarised in Table 1

Method	Bioindicator	Environment	Metric	Reference	Related studies
Physicochemical and biological	Phytoplankton, macroinvertebrates, diatoms, algae,	Biota, water, sediment	Berger-Parker, dominance, evenness, abundance, Shannon, Margarlef’s, diatom biological index	1, 2, 3, 6, 7, 9, 10, 11, 12	Bellinger and Sigee (2015); Lobo et al. (2016); Forio and Goethals (2020)
Chemical and biological	Algae, phytoplankton	Biota, water, sediment	Enrichment factor, pollution load index, contamination factor, index of geo-accumulation, abundance, richness	4, 8, 16, 19, 21	Torrisi et al. (2010); Ismail and El Zokm (2023)
Biological	Algae, bacterial DNA (16S RNA), antibiotic-resistant genes	Biota, water, sediment	Abundance, diversity	13, 14, 15,16, 17, 18, 20	Apothéloz-Perret-Gentil et al. (2021)

Integrating molecular techniques in biomonitoring is a potential approach to revolutionise aquatic pollution assessment (Li et al. 2010; Lobo et al. 2016). In this review, Laffite et al. (2020) observed a significant correlation between metals and 16 s rRNA, suggesting a close link between metal pollution and human-mediated pressures on an urban river in the Democratic Republic of Congo. Pereira-da-Conceicoa et al. (2021) demonstrated the relevance of integrating environmental DNA (eDNA) into existing monitoring metrics to provide additional taxonomic resolution for aquatic biodiversity management in South African streams. The application of molecular methods has also been observed to substantially improve the biomonitoring of streams in France, China, and Switzerland compared to the traditional morphotaxonomic methods (Apothéloz-Perret-Gentil et al. 2021; Keck et al. 2018; Li et al. 2018). However, Perry et al. (2022) noted a significant drawback in the integration of eDNA principally inhibited by inadequate reference data for SSA in the gene banks. The lack of reference eDNA databases, downstream transport, dilution of DNA fragments, and introduction of terrestrial DNA, among other challenges, has also been observed in other regions, e.g., Finland (Norros et al. 2022), Switzerland (Deiner et al. 2016), Canada (Laporte et al. 2022), and globally (Beng and Corlett 2020).

### Integrating microalgae into emerging technologies for monitoring metal pollution in stream ecosystems

Several cutting-edge emerging technologies are gaining popularity as complementary approaches to support conventional monitoring and assessments of stream ecosystems, as described below.

#### Microalgal-eDNA metabarcoding

Based on the current review, recently, limited studies have incorporated microalgal eDNA in biomonitoring aquatic metal pollution in the SSA. For instance, Laffite et al. (2020) investigated the co-contamination and seasonal variability of metal in bed sediments of urban rivers in DRC using bacterial eDNA. Significant correlations were observed between metal concentrations and 165 s rRNA bacterial densities, linking pollution to anthropogenic inputs. In South Africa, Jordaan et al. (2019), using the 16 s rRNA gene profiles, noted a substantial impact of pH and metal contamination from mining on bacterial diversity and community structure in the lower Wonderfonteinspruit catchment rivers. Furthermore, Perry et al. (2022) demonstrated the cost–benefit of using bulk samples and eDNA for multispecies biodiversity monitoring of Namibia’s freshwater systems. However, in most SSA countries, few studies, if any, have integrated microalgal eDNA in aquatic metal pollution biomonitoring. Given the sparsity of eDNA biomonitoring research data in SSA, more effort is needed to develop methods adapted to regional and local conditions and to generate eDNA gene-bank reference data to increase our understanding of SSA aquatic ecosystems (Perry et al. 2022). In addition, the performance of eDNA tools in biomonitoring aquatic metal pollution in SSA lotic ecosystems compared with the conventional monitoring approaches is not adequately investigated. Therefore, further research is needed to address this methodological gap by integrating microalgae-based eDNA biomonitoring of aquatic metal(loid) pollution at the community, species, and molecular level in stream ecosystems of SSA (Stat et al. 2017).

In the recent past, most of the eDNA biomonitoring has been conducted in the global North (Resh 2007). For instance, Cilleros et al. (2019) compared the effectiveness of eDNA metabarcoding and conventional morphotaxonomic techniques while assessing the diversity of fish assemblages in 38 streams of the French Guiana. Their findings revealed that while traditional taxonomic methods offered a more comprehensive inventory of fish taxa, they were spatially limited. In contrast, eDNA metabarcoding, when complemented with classical methods, was a more comprehensive and efficient approach for rapidly assessing and monitoring fish diversity on a larger spatial scale. Similarly, Gleason et al. (2021) conducted a study in southern Ontario, Canada, comparing eDNA metabarcoding techniques with traditional kick-net sampling to monitor lotic macroinvertebrate communities. Their findings demonstrated that eDNA techniques, especially metabarcoding of bulk tissues, provided a better representation of the diversity of macroinvertebrate taxa at a finer spatial resolution than traditional methods. However, in SSA, few studies have integrated eDNA in aquatic biomonitoring of metal pollution (e.g., Laffite et al. 2020; Jordaan et al. 2019; Perry et al. 2022). Therefore, progressive regional research must be strengthened to overcome the current limitations of aquatic eDNA biomonitoring, such as inadequate e-DNA reference data (Perry et al. 2022).

While acknowledging that eDNA is more appropriate for short-term monitoring, eDNA data can be used integratively with long-term monitoring approaches, such as remote-sensing, biosensor, and citizen science (Hansen et al. 2020). For instance, eDNA data can be used to validate or ground-truth remotely sensed data to ensure the reliability of long-term monitoring systems. Additionally, the integration of eDNA can increase the resolution of pollutant detection at sub-lethal and ensure the validity and consistency of sensed data.

#### Biosensor systems for aquatic biomonitoring

Recently, a variety of biosensors gained high attention and have been employed in in-situ for real-time monitoring and detection of environmental contaminants (Huang et al. 2023). A biosensor typically comprises a biosensing probe and a transducer that detects a contaminant by producing a quantifiable signal (Mishra et al. 2019; Rovira and Domingo 2019). The biosensor probe material can be antibody-, DNA-, whole-cell-, or enzyme-based (Singh et al. 2020). The transducer translates the biological signals to optical or electrical signals via optical, physicochemical, or piezoelectric material (Nguyen et al. 2019). Electrochemical and optical biosensors have been employed to detect and quantify metals, including Hg^+^, Pb^2+^, Zn^2+^, Cu^2+^, and Cd^2+^ in water (Wu et al. 2023). Advances in nanotechnology have further improved the performance of biosensors due to the numerous benefits of larger sensing equipment. Nanosensor materials improve biosensor efficiency for colour sensing, target sensitivity, and carrier capacity. Additionally, nanomaterials have high thermal and electrical conductivity (Huang et al. 2021; Abdel-Karim 2024).

Whole-cell microbial biosensors detect metal ions based on the genetic element that responds to target metals (Huang et al. 2023). In aquatic environments, whole-cell bacterial biosensors have been used to detect bioavailable metals with high sensitivity (Cerminati et al. 2015). Alfadaly et al. (2021) applied a complementary target resistive *Rhizobium* bacteria-based and *Rhodotorula* fungi-based bioelectrochemical sensor to detect and remove Cr^6+^ and Cd^2+^ ions from polluted water. The bacterial component exhibited superior performance for metal resistivity and removal. In another study, Cerminati et al. (2015) confirmed the efficacy of a broad-spectrum whole-cell-based metal biosensor as a screening tool for the presence of bioavailable Au, Hg, Pb, and Cd in water.

Genetically engineered DNA-based microbial biosensors combined with electrochemical transducers broaden the applicability of cell-based biosensors for early monitoring and detection of metal ions in water Jeon et al. (2022). According to Jeon et al. (2022), the mutation of a regulatory protein ZntR in *Escherichia coli* enhanced the selectivity of Pb^2+^ ions after metal ion-exporting genes were deleted in the host cells. Furthermore, Nourmohammadi et al. (2020) observed high specificity for Pb^2+^ bacterial biosensor expressing a luciferase reporter gene controlled by *pbr*/*cadA* promoters in *Cupriavidus metallidurans* in a genetically engineered bacterial system.

According to Huang et al. (2023), biosensors are low-cost, easy to use, and energy-saving and require minimal pre-sample treatment. In addition, biosensor technology uses non-hazardous materials and has considerably low carbon footprints compared to physicochemical methods. Furthermore, the integration of bacteria into biosensor technologies offers numerous benefits in the detection and monitoring of aquatic metal pollution. Biosensors and microalgae serve as complementary tools, offering different perspectives and capabilities. Biosensors enhance the detection and quantification of metal contaminants in real-time or near real-time, thereby allowing for rapid detection and tracking of metal pollution (Wu et al. 2023).

In contrast, microalgae are reliable bioindicators of long-term exposure to metal pollution, reflecting the historical trend. A combination of biosensor data with microalgae assessments reflects a comprehensive understanding of short and long-term metal pollution dynamics in impacted streams. Additionally, biosensors often exhibit high sensitivity and specificity for detecting target metal ions, enabling the detection of sub-lethal metal concentrations (Huang et al. 2023). Microalgae, while sensitive to metal pollution, may not always provide precise measurements of metal concentrations at low levels or in complex environmental matrices. The integration of microalgal DNA into biosensors has the advantage of sensitivity and specificity, especially in natural environments with multi-elemental metal contaminants. Furthermore, biosensors are robust and can simultaneously be deployed at multiple locations within stream ecosystems. This spatial advantage complements the localised application of microalgae per time to monitor metal contamination.

Additionally, biosensors are often portable and easy to deploy, making them accessible for field-based monitoring in remote or challenging environments. However, the high cost and technological requirements of nanomaterials production could impede the production and application of nanobiosensors, particularly in developing countries.

#### Remote sensing

Satellite-based remote sensing (RS) and hyperspectral imaging is a cost-effective monitoring approach that enhances extensive and rapid spatial coverage of the Earth’s surface with repeatability capabilities for investigating environmental systems (Reddy 2018; Pettorelli et al. 2018). By detecting unique spectral signatures of various substances, including metals, RS can pinpoint the presence and concentration of specific metals in waters via hyperspectral electromagnetic radiation. For instance, Lin et al. (2024) determined the concentration of metals in China’s Dalian Lake using hyperspectral analysis and genetics algorithms. The integration of RS techniques with biosensor data capture probes also improved the spatial mapping of metals and sediments along Egypt’s Red Sea Coast (Mohammed et al. 2024). Bresciani et al. (2016) mapped patterns of cyanobacterial blooms in five Italian lakes using a suite of aerial and space-borne hyperspectral sensors with increased accuracy. Guo et al. (2022) and Cao et al. (2018) integrated RS to model metals and chlorophyll-*a* concentrations in water. The models provided high retrieval accuracy and realistic information.

Data generated from RS enables the creation of detailed spatiotemporal maps of aquatic metal pollution and hotspots mapping. Integrating RS data with water quality measurements and GIS data provides a comprehensive understanding of metal pollution dynamics in aquatic ecosystems (Yu et al. 2020; Zhu et al. 2022). Furthermore, RS is cost-effective and efficient compared to traditional field-based methods, enabling the rapid collection of extensive data over large areas with lower monitoring costs (Avtar et al. 2020). Overall, RS serves as a valuable tool in monitoring metal pollution in aquatic environments, providing timely and spatially explicit information crucial for informed decision-making and effective environmental management strategies.

The complementarity between RS and microalgae in monitoring metal pollution in aquatic systems is multifaceted. RS provides wide spatial coverage, allowing for the monitoring of large water bodies and the identification of metal pollution hotspots (Chi et al. 2016). However, RS may lack the spatial resolution needed to detect localised pollution events or variations. Microalgae, on the other hand, can be highly sensitive to rapid changes in metal concentrations and hence reflect localised pollution impacts. Furthermore, RS allows for the monitoring of changes in metal pollution over time by capturing images at different intervals. In contrast, the rapid response of microalgae to changes in metal concentrations makes useful indicators of short-term pollution events.

RS data can be validated and calibrated using ground-truthed data, including responses from bioindicators, including microalgae (Cook et al. 2023). This process enables researchers can assess the accuracy of remote-sensing-derived metal pollution estimates and refine remote-sensing algorithms to improve their reliability. Microalgae responses to metal pollution can serve as early warning indicators of environmental degradation. With a combination of RS data with real-time monitoring of microalgae populations, researchers can develop early warning systems to alert authorities to potential pollution events or ecosystem stressors, enabling timely intervention and mitigation efforts.

#### Citizen science

Citizen science monitoring involves volunteers (i.e., mainly non-professionals), often the riparian communities, and is fundamentally public participation by stakeholders in environmental stewardship (Moharana 2021; Fraisl et al. 2022). Citizen science has the potential of upscaling field studies to a regional or global extent coupled with centralised monitoring efforts that enhance extensive and well-coordinated environmental monitoring, which can produce large datasets rapidly. Miguel-Chinchilla et al. (2019) analysed citizen-sensed catchment data on stream turbidity which contributed nearly 12% value to the study. Babiso et al. (2023) analysed water quality data collected from the Meki River, Ethiopia, by citizen scientists. The study results indicated a good agreement with selected parameters, which implied the accuracy of citizen-collected data. Additionally, Thornhill et al. (2017) used citizen science stream data from the metropolis of China to model and classify predictors of water quality using random forest models with reliable results.

The incorporation of smartphone technology to measure and record environmental data under the citizen science programs has greatly improved the speed, volume, and quality of data. Malthus et al. (2020) examined the impact of citizen science smartphone applications (Apps) on remotely sensed surface reflectance, stream sediment, and algal concentrations in 32 stream sites in eastern Australia. Smartphone Apps provide a friendly interface for citizen scientists to engage with and use sophisticated modern water quality monitoring technology. Smartphones are widely accessible, and the Apps are customised for objective, comprehensive, and accurate data capture (Pattinson et al. 2023).

Citizen science can complement microalgal biomonitoring of aquatic metal pollution in stream ecosystems through increased data collection. Citizen science projects engage a broader range of participants, allowing for more extensive data collection across various locations and times (Njue et al. 2019; Babiso et al. 2023). This can provide a more comprehensive understanding of the spatial and temporal dynamics of metal pollution in stream ecosystems. Furthermore, citizen science involves community engagement and creates social accountability and awareness towards environmental stewardship (Ruppen and Brugger 2022). Involving citizens in scientific monitoring fosters a sense of ownership and stewardship over local environments. This leads to increased awareness of environmental issues such as metal pollution and promotes sustainable behaviours to mitigate them. Citizen science, being a cost-effective metal pollution monitoring technique, can leverage the manpower and resources of volunteers, reducing the costs associated with monitoring efforts (Njue et al. 2019; Ruppen and Brugger 2022). This enables more frequent sampling and monitoring, which is essential for detecting changes in metal pollution levels over time. However, there is a need to identify and address the potential for errors and biases in integrating this approach in the biomonitoring of stream ecosystems (Follett and Strezov 2015).

### Challenges and opportunities in integrating microalgae in aquatic biomonitoring of metal pollution

Despite the bottlenecks in the integrative monitoring of aquatic metal pollution in lotic systems of SSA using algal communities as bioindicators, several opportunities also present further room for developing a microalgae-based assessment of stream health status in the region. We highlight the challenges and opportunities for developing higher resolution, site-specific, and species-targeted microalgal-based bioassessment in SSA.

#### Challenges

From our literature search, while other bioindicator taxa, particularly macroorganisms, are popular options, the use of algal communities to assess aquatic metal pollution, where this has been attempted, has been limited to the morphotaxonomic level. Besides not offering the benefit of higher resolution in detecting sub-lethal metal contamination, the absence of region-specific baseline data in several SSA sub-regions further limits the comprehensiveness of their use as bioindicators. Additionally, accurate identification of microalgal species requires trained morphotaxonomists and special equipment, such as the high-resolution scanning electron microscope, which may be limited in SSA. The uptake and integration of microalgae-based bioindicators into cutting-edge biomonitoring tools such as molecular (eRNA and eDNA), artificial intelligence systems (e.g., biosensors), and geospatial systems are yet to take off significantly. Accurate identification is essential for proper assessment and quantification of the magnitude of metal pollution and potential ecological risks to the provision of stream ecosystem services in SSA.

Microalgal communities and populations exhibit significant seasonal fluctuations, which must be understood to allow the partitioning of metal-pollution-induced impacts. Climate change, seasonality, and natural and anthropogenic factors influence microalgal community composition and species abundance. Seasonal fluctuations, such as variations in rainfall and temperature, significantly influence the hydrology of aquatic ecosystems, affecting the transport and deposition of metals (Maphanga et al. 2024). For instance, intense rainfall during the wet season can remobilise metals from soil and sediment into water bodies, causing elevated metal concentrations (Conrad et al. 2020). Conversely, dry seasons may concentrate metals due to decreased dilution and increased evaporation rates (Edokpayi et al. 2017). Extreme climatic events, such as floods and drought, further exacerbate metal pollution by altering metal transport and sedimentation patterns, which may introduce metals from repositories into water bodies (Xia et al. 2015; Wijngaard et al. 2017). Anthropogenic activities such as mining, industrial effluents, and agricultural runoff are significant sources of metal pollution in sub-Saharan Africa (Laffite et al. 2020). These activities introduce high concentrations of metals such as Pb, Hg, Cu, and Cd into aquatic ecosystems, which are highly toxic to aquatic biota (Hama Aziz et al. 2023; Fatmi et al. 2023). The interaction between seasonal dynamics, extreme climatic events, and anthropogenic activities underscores the complexity of integrating microalgae in monitoring aquatic metal pollution in SSA.

Furthermore, in the aquatic environment, metal species and mixtures interact differently with microalgae community species and vary in toxicity. Understanding these interactions and the ecological impact on microbial bioindicators, including microalgae, in the SSA is poorly understood due to the complexity and, hence, the need for cutting-edge research using advanced methods and sophisticated analytical equivalents. Unfortunately, many regions in SSA suffer from limited monitoring infrastructure, which hampers effective biomonitoring and data availability. Also, SSA, as a low to middle-income subregion, is marked by limited financial capacity to fully support advanced environmental stewardship programs in light of other “critical” financial obligations.

#### Opportunities

Similar to other tropical regions, SSA has a high diversity of microalgal communities and species richness due to its varied and extensively interconnected aquatic habitats. The biodiversity and richness can be leveraged to select the highly sensitive and most indicative species that respond specifically to individual and mixtures of aquatic metal pollution at sub-lethal concentrations. Since the most suitable microalgal communities and species have high sensitivity to changes in water quality, they can serve as potential early warning indicators of aquatic metal pollution episodes in river systems. Therefore, integrating microalgae in stream health assessment will improve early detection and further inform proactive interventions in managing stream ecosystems in the SSA mining regions.

Collaborative research within the SSA and with international research institutions and partners will address the limitations of access to advanced analytical tools, expertise, and limited funding. In particular, adopting modern research and monitoring tools such as eDNA and molecular biosensors will improve the resolution of detection of aquatic metal pollution. Establishing and expanding baseline data collection and accessible online databases will further accelerate the integration of microalgae for monitoring aquatic metal pollution in aquatic ecosystems across SSA.

## Conclusion and future perspectives

Integrating microalgal communities as bioindicators of aquatic metal pollution in rivers of sub-Saharan Africa holds great promise for enhancing water quality monitoring and environmental conservation efforts. However, in the past decade and a half, the inclusion of microalgal taxa for integrative monitoring into aquatic metal pollution monitoring programs in the SSA has been low but gradually improving past 2020. The region is still lagging in the integration of emerging tools, such as environmental DNA, and technological advances, such as artificial intelligence models, remote sensing, and citizen science, that offer potential benefits of high precision, speed, reduced costs, and eco-friendly green technologies in monitoring and assessment of stream ecosystem health across its mining landscapes.

Despite the lack of a standardised, synchronised, and adequately documented microalgal database, inadequate microalgal taxonomic and molecular assessment expertise, limited monitoring and processing infrastructure, and economic constraints, the integrative microalgae-based approach offers significant opportunities for addressing aquatic metal pollution in the SSA lotic ecosystems. The high biodiversity of microalgae in SSA presents a vast pool for selecting suitable site-specific (sensitive and indicative) taxa that respond specifically to individual and mixtures of metal pollutants. Different microalgal taxa have been combined with other bioindicator groups to increase the sensitivity to pollutant detection source tracking and quantification. This uniqueness emphasises the central role of microalgae in aquatic ecosystem biomonitoring initiatives. Moreover, the rapid response to changes in water quality during integrative monitoring positions microalgae as potential early warning indicators for aquatic metal pollution events that impact aquatic ecosystems.

Fundamentally, there is an urgent need to prioritise efforts to institutionalism and strengthen and standardise national and regional baseline data collection on microalgae dynamics in response to metal pollution in aquatic ecosystems across SSA. Such data will serve as a foundation for accuracy and a reference point for improving the assessments of metal pollution and ecological impacts on the region’s aquatic ecosystems.

Collaboration among regional and international research institutions and organisations can lead to the development of integrated monitoring networks. These networks can leverage advanced analytical tools, technologies, and expertise to enhance data collection and analysis. Furthermore, involving riparian communities in data collection and monitoring efforts fosters a sense of collective responsibility and ownership of the stream ecosystem resources. In fact, “citizen science” initiatives are crucial to empowering riparian communities to participate actively in the conservation of stream ecosystems.

Continued research into the interactions between metal pollutants and microalgal species is essential for better understanding the ecological consequences of aquatic metal pollution. Complementing conventional monitoring with innovative techniques, such as artificial intelligence, molecular tools, and remote sensing, must be prioritised to improve the overall efficiency and maximise the productivity of environmental stewardship in metal mining regions.

## Data Availability

All the data generated are available in this review. Additional data and information can be sourced from the cited references and online databases or sources.
